# Evaluation of ChatGPT-4’s Performance in Therapeutic Decision-Making During Multidisciplinary Oncology Meetings for Head and Neck Squamous Cell Carcinoma

**DOI:** 10.7759/cureus.68808

**Published:** 2024-09-06

**Authors:** Kenza Alami, Esther Willemse, Marie Quiriny, Samuel Lipski, Celine Laurent, Vincent Donquier, Antoine Digonnet

**Affiliations:** 1 Otolaryngology, Jules Bordet Institute, Bruxelles, BEL; 2 Surgical Oncology, Jules Bordet Institute, Bruxelles, BEL; 3 Otolaryngology - Head and Neck Surgery, Hôpital Ambroise-Paré, Mons, BEL; 4 Otolaryngology - Head and Neck Surgery, Hôpital Universitaire de Bruxelles (HUB) Erasme Hospital, Bruxelles, BEL

**Keywords:** artificial intelligence chatgpt-4, ent - ear nose and throat, head and neck squamous cell carcinoma (hnscc), multidisciplinary oncologic meeting, oral oncology

## Abstract

Objectives

First reports suggest that artificial intelligence (AI) such as ChatGPT-4 (Open AI, ChatGPT-4, San Francisco, USA) might represent reliable tools for therapeutic decisions in some medical conditions. This study aims to assess the decisional capacity of ChatGPT-4 in patients with head and neck carcinomas, using the multidisciplinary oncology meeting (MOM) and the National Comprehensive Cancer Network (NCCN) decision as references.

Methods

This retrospective study included 263 patients with squamous cell carcinoma of the oral cavity, oropharynx, hypopharynx, and larynx who were followed at our institution between January 1, 2016, and December 31, 2021. The recommendation of GPT4 for the first- and second-line treatments was compared to the MOM decision and NCCN guidelines. The degrees of agreement were calculated using the Kappa method, which measures the degree of agreement between two evaluators.

Results

ChatGPT-4 demonstrated a moderate agreement in first-line treatment recommendations (Kappa = 0.48) and a substantial agreement (Kappa = 0.78) in second-line treatment recommendations compared to the decisions from MOM. A substantial agreement with the NCCN guidelines for both first- and second-line treatments was observed (Kappa = 0.72 and 0.66, respectively). The degree of agreement decreased when the decision included gastrostomy, patients over 70, and those with comorbidities.

Conclusions

The study illustrates that while ChatGPT-4 can significantly support clinical decision-making in oncology by aligning closely with expert recommendations and established guidelines, ongoing enhancements and training are crucial. The findings advocate for the continued evolution of AI tools to better handle the nuanced aspects of patient health profiles, thus broadening their applicability and reliability in clinical practice.

## Introduction

In the dynamic realm of medical science, especially in oncology fields, therapeutic decision-making embodies a complex amalgam of patient-specific considerations and evolving evidence-based practices. This complexity is importantly observed in head and neck squamous cell carcinomas (HNSCC), where treatment decisions must meticulously balance both the eradication of malignancies with the preservation of essential functions and quality of life. This is while controlling comorbidities and any indications for prophylactic enteral nutrition [1]. Multidisciplinary oncology meetings (MOMs) are significant in this area, providing a collaborative platform for comprehensive case evaluation and consensus-driven management strategies [2]. Medical knowledge is constantly evolving [3,4], and the available emergence of artificial intelligence (AI) and deep learning in the past decade has introduced new tools that may significantly enhance the healthcare field [5] if proven reliable. Thus, AI development, paced by innovations such as OpenAI’s ChatGPT-4 (Open AI, ChatGPT-4, San Francisco, USA), has enabled new capacities to factor in medical decision-making and healthcare [6]. Launched on November 20, 2022, ChatGPT-4 uses advanced algorithms, native language processing, and machine learning to provide fast, user-friendly, and insightful answers [7,8]. Its current database was last updated up to April 2023. In fact, guidelines from esteemed societies such as the National Comprehensive Cancer Network (NCCN), the American Society of Clinical Oncology (ASCO), and the European Society for Medical Oncology (ESMO) are used as decisive resources by ChatGPT-4. These guidelines, frequently updated through newly published evidence and expert consensuses, aim to reduce inconsistencies in care and optimize patient outcomes. The dynamic nature of medical research needs constant adaptations and specified applications of these guidelines while applied in clinical practice. Thus, its ability to access extensive knowledge databases, including those provided by NCCN, ASCO, ESMO, UpToDate, Medscape, and PubMed, has positioned ChatGPT-4 as a potentially crucial tool for clinical practitioners [9]. Therefore, AI development presents a unique opportunity. Although its application in healthcare has generated significant interest and debate [10,11], using an AI like ChatGPT-4 as a new tool in clinical settings, especially in HNSCC management, would be striking to explore. Hence, this study intends to critically assess ChatGPT-4 value in therapeutic decision-making for patients with head and neck carcinomas. We aim to objectively compare therapeutic recommendations provided by ChatGPT-4 for HNSCC treatments with first those assessed in MOM of Jules Bordet Institute and secondly with NCCN recommendations. By looking into the consistencies, divergences, and reliabilities of AI-supported clinical decision-making with established multidisciplinary consensus, this research aspires to enlighten AI’s capacities in improving oncological care.

## Materials and methods

Study setting

The study was approved by the institutional ethics committee at the Jules Bordet Institute, Belgium (reference: CE3827). This exploratory retrospective study reviewed 1,325 patients discussed at the MOM between January 1, 2016, and December 31, 2021. We selected 263 patients with squamous cell carcinoma of the oral cavity, oropharynx, hypopharynx, or larynx.

Inclusion criteria included histologically confirmed squamous cell carcinoma of the oral cavity, oropharynx, hypopharynx, or larynx; pretreatment CT scan, MRI, or PET-CT; esogastroscopy; full follow-up at the Jules Bordet Institute; and availability of necessary data.

Necessary data included year of diagnosis, sex, age at diagnosis, WHO performance status, smoking and alcohol history, American Society of Anesthesiologists (ASA) score, renal clearance, ejection fraction, BMI, Nutritional Risk Screening (NRS) score, Nutritional Risk Index (NRI) score, G8 score if applicable, tumor location (oral cavity, oropharynx, hypopharynx, or larynx), cTNM stage, and P16 status if applicable. Adjuvant treatment, local recurrence, and metastases outside the head and neck area within two years of the first diagnosis were also recorded.

Potential therapeutic decisions were categorized as percutaneous endoscopic gastrostomy (PEG), exclusive radiotherapy, chemotherapy, radio-chemotherapy, immunotherapy (IT), upfront surgery (US), salvage surgery (SS), lymph node dissection (LND), sentinel lymph node (SLN) biopsy, and comfort care (CC).

Exclusion criteria included lack of identifiable decision during the MOM; missing necessary data; patient refusal to use their data; patients with a known oncologic history; or patients with synchronous neoplasms.

ChatGPT-4 performance

**Figure 1:**
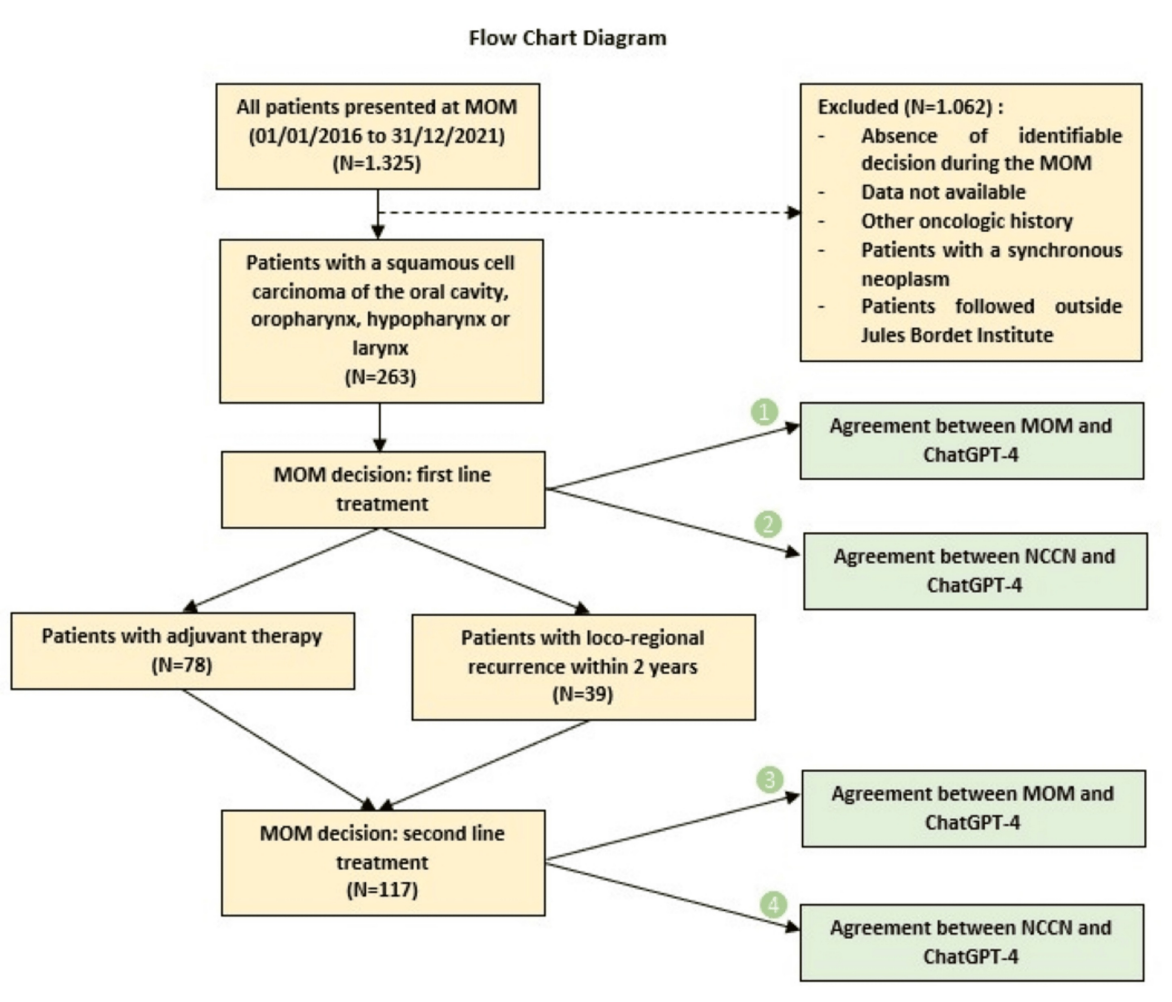
Flowchart

Patient data with complete information were presented to ChatGPT-4 (OpenAI, San Francisco, USA), which was systematically queried to propose potential therapeutic regimens. For instance, in 2019, a 54-year-old patient with squamous cell carcinoma of the mobile tongue, clinical stage T3N1M0, and a history of chronic alcohol and tobacco use, had a WHO performance score of 0, ASA II, BMI of 20.2, NRS score of 3, and an NRI score of 79, with no cardiac or renal failure. ChatGPT-4 was asked to propose a first-line treatment and to determine whether an immediate gastrostomy was warranted. Postoperatively, the patient had undergone surgery and gastrostomy, with findings including invasive squamous cell carcinoma, well-differentiated (keratinizing), measuring 4.5 cm in size and 1.5 cm in thickness, with perineural invasion and vascular emboli. There was focal invasion at the deep margin, with corresponding healthy cuts, and two out of 42 lymph nodes (level III right neck dissection) were invaded, without capsular invasion. The UICC 8th edition stage was pT3N2b. ChatGPT-4’s recommendations were collected and compared with the propositions from the multidisciplinary oncologic meetings (MOMs) and the NCCN recommendations for the relevant year (Figure 1) [12-16].

Statistical analysis

Statistical analyses were conducted using the Statistical Analysis System (SAS). Treatments proposed by the MOM, the NCCN guidelines, and ChatGPT-4 were assigned predefined numerical codes in a matrix, facilitating the evaluation of consistency between MOM and ChatGPT-4, as well as NCCN and ChatGPT-4, through kappa analysis. The Kappa method assesses the degree of agreement between two evaluators relative to chance. The Kappa coefficient (K) is interpreted as follows [17]: -0 indicates less than chance agreement; 0.01-0.20 represents slight agreement; 0.21-0.40 signifies fair agreement; 0.41-0.60 denotes moderate agreement; 0.61-0.80 indicates substantial agreement; and 0.81-1 reflects almost perfect agreement.

## Results

Subjects and setting

Of the 1,325 patients discussed in MOM between January 1, 2016 and December 31, 2021, the clinical history of 263 patients with squamous cell carcinoma of the oral cavity (32.7%), oropharynx (30%), hypopharynx (15.2%), or larynx (22.1%) was completed and presented to ChatGPT-4. There were 57 females (21.7%) and 206 males (78.3%). The mean age of patients was 63 ± 10.6 years.

Treatment performance

Agreement Between MOM and ChatGPT-4 for First-Line Treatment

We examined the agreement between the treatment recommendations made by ChatGPT-4 and those provided during MOMs for 263 patients newly diagnosed with squamous cell carcinoma of the oral cavity, oropharynx, hypopharynx, or larynx. Notably, no indication for first-line IT was given to any of the 263 patients. Within this framework, the only noteworthy recommendation pertains to CC, which showed an almost perfect agreement (Kappa score of 0.93) (Table 1). Also, the overall agreement between ChatGPT-4 and the MOMs for first-line treatment suggests a fair agreement (simple Kappa coefficient: 0.35 with a 95% CI of 0.28-0.42).

**Table 1:** ChatGPT-4: MOM sensitivity, specificity, and agreement at first-line treatment CC, comfort care; LND, lymph node dissection; MOM, multidisciplinary oncology meeting; PEG, percutaneous endoscopic gastrostomy; SLN biopsy, sentinel lymph node; US, upfront surgery

	PEG	Radiotherapy	Chemotherapy	Radio-chemotherapy	US	LND	SLN biopsy	CC	Overall
Sensitivity	80%	54%	50%	90%	57%	43%	62%	100%	67%
Specificity	66%	94%	98%	66%	94%	97%	95%	100%	89%
Kappa score	0.47	0.53	0.28	0.5	0.52	0.45	0.44	0.93	0.35
95% CI	0.36-0.57	0.39-0.67	-0.88	0.4-0.6	0.42-0.61	0.34-0.55	0.22-0.65	0.8-1	0.28-0.42

With the exclusion of the “gastrostomy” factor, the overall agreement between ChatGPT-4 and the MOMs for first-line treatment improved, with the Kappa score increasing from 0.35 to 0.48 (95% CI: 0.4-0.55). Thus, we are shifting from a fair agreement to a moderate agreement.

Agreement Between NCCN and ChatGPT-4 for First-Line Treatment

For the 263 patients included in our study, we evaluated the agreement between ChatGPT-4 and the NCCN guidelines according to the year the patient was managed. All recommendations made in the MOMs were consistent with the NCCN guidelines. We found a substantial agreement for radiotherapy, chemotherapy, radio-chemotherapy, US, LND, and SLN biopsy, with respective Kappa scores of 0.78, 0.8, 0.67, 0.72, 0.65, and 0.8 (Table 2). Furthermore, there was an almost perfect agreement for CC, with a Kappa score of 1 (Table 2). Additionally, the overall agreement between ChatGPT-4 and the NCCN guidelines for first-line treatment demonstrated a substantial agreement (simple Kappa coefficient: 0.72 with a 95% CI of 0.66-0.79).

**Table 2:** ChatGPT-4: NCCN sensitivity, specificity, and agreement at first-line treatment CC, comfort care; LND, lymph node dissection; NCCN, National Comprehensive Cancer Network; SLN, sentinel lymph node; US, upfront surgery

	Radiotherapy	Chemotherapy	Radio-chemotherapy	US	LND	SLN biopsy	CC	Overall
Sensitivity	70%	100%	100%	72%	59%	100%	100%	86%
Specificity	100%	99%	74%	99%	100%	98%	100%	96%
Kappa score	0.78	0.8	0.67	0.72	0.65	0.8	1	0.72
95% CI	0.68-0.89	0.41-1	0.58-0.75	0.64-0.8	0.55-0.74	0.65-0.96	1-1	0.66-0.79

Agreement Between MOM and ChatGPT-4 for Second-Line Treatment

Among the 263 patients included, 117 either received adjuvant treatment or experienced loco-regional recurrence of their cancer within two years (without receiving adjuvant treatment). Therefore, for those 117 patients for whom a second-line treatment was proposed, we compared the management proposed by the MOMs and by ChatGPT-4. We observed almost perfect agreement for the recommendations on chemotherapy, SS, LND, and CC, with respective Kappa scores of 0.85, 0.86, 0.83, and 1 (Table 3). Moreover, there was substantial agreement for the recommendations on radio-chemotherapy and IT, with respective Kappa scores of 0.71 and 0.74 (Table 3).

**Table 3:** ChatGPT-4: MOM sensitivity, specificity, and agreement at second-line treatment CC, comfort care; IT, immunotherapy; LND, lymph node dissection; MOM, multidisciplinary oncology meeting; PEG, percutaneous endoscopic gastrostomy; SS, salvage surgery

	PEG	Radiotherapy	Chemotherapy	Radio-chemotherapy	SS	LND	CC	IT	Overall
Sensitivity	79%	59%	100%	90%	83%	84%	100%	75%	84%
Specificity	58%	90%	99%	84%	99%	97%	100%	99%	91%
Kappa score	0.28	0.52	0.85	0.71	0.86	0.83	1	0.74	0.56
95% CI	0.06-0.5	0.34-0.69	0.57-1	0.58-0.84	0.75-0.97	0.66-0.99	1-1	0.4-1	0.32-0.8

However, the overall agreement between ChatGPT-4 and the MOMs for second-line treatment suggests a moderate agreement (simple Kappa coefficient: 0.56 with a 95% CI of 0.32-0.8).

With the exclusion of the “gastrostomy” factor, the overall agreement between ChatGPT-4 and MOM for second-line treatment improved, with the Kappa score increasing from 0.56 to 0.78 (95% CI of 0.64-0.92). Thus, we are shifting from a moderate agreement to a substantial agreement.

Agreement Between NCCN and ChatGPT-4 for Second-Line Treatment

We assessed the agreement between ChatGPT-4 and the NCCN guidelines based on the year of second-line treatment management for 117 patients (either adjuvant treatment or treatment for loco-regional recurrence within two years). We observed almost perfect agreement for chemotherapy, radio-chemotherapy, SS, LND, CC, and IT, with respective Kappa scores of 1, 0.83, 0.95, 0.96, 1, and 0.85) (Table 4). There was also substantial agreement for the indications of radiotherapy, with a Kappa score of 0.77 (Table 4). Additionally, the overall agreement between ChatGPT-4 and the NCCN guidelines for second-line treatment indicated substantial agreement (simple Kappa coefficient: 0.66 with a 95% CI of 0.56-0.76).

**Table 4:** ChatGPT-4: NCCN sensitivity, specificity, and agreement at second-line treatment CC, comfort care; IT, immunotherapy; LND, lymph node dissection; NCCN, National Comprehensive Cancer Network; SS, salvage surgery

	Radiotherapy	Chemotherapy	Radio-chemotherapy	SS	LND	CC	IT	Overall
Sensitivity	79%	100%	93%	97%	100%	100%	75%	92%
Specificity	95%	100%	92%	99%	97%	100%	100%	98%
Kappa score	0.77	1	0.83	0.95	0.96	1	0.85	0.66
95% CI	0.64-0.9	1-1	0.73-0.94	0.89-1	0.88-1	1-1	0.57-1	0.56-0.76

Lastly, in total, our study identified 52 patients for whom ChatGPT-4’s management recommendations differed from those of the MOM as well as the NCCN guidelines for the relevant year.

## Discussion

The utilization of ChatGPT-4 has been examined in various studies that gauge its efficacy in aiding diagnostic processes, devising treatment strategies, and even within the scope of medical education. Investigations such as those conducted by Kung et al. [18] have demonstrated that ChatGPT-4 can match healthcare professionals on standardized exams like the United States Medical Licensing Examination, showcasing its substantial capability to comprehend and manipulate complex medical information. In otolaryngology, currently, there are less than 10 studies probing ChatGPT-4’s potential, with none addressing its role in multidisciplinary oncological decision-making. As far as we are aware, this study is the inaugural one to evaluate ChatGPT-4’s efficacy in multidisciplinary oncological practice with the presentation of real clinical cases to the chatbot.

In Kuşcu et al.’s research [19] regarding ChatGPT-4’s ability to answer questions screened through social media posts about various topics related to head and neck cancers (such as diagnosis, treatment, and operative risks), the results are similar to ours: ChatGPT-4 yielded “comprehensive/correct” answers to 133 out of 154 questions (86.4%), while the rates of “incomplete/partially correct” and “mixed with accurate and inaccurate data/misleading” responses were 11% and 2.6%, respectively. The model provided correct answers for 88.9% of the treatments for head and neck cancers, whereas in our study, overall treatment accuracy ranges from 67% to 92%. However, such performance levels do not consistently translate to practical clinical application, where decision-making is often influenced by multidimensional factors, absent in a testing setting [19].

The theoretical performance of ChatGPT-4 in otolaryngology has been newly validated by two original studies. In particular, Chiesa-Estomba et al. observed significant agreement (p < 0.026) between ChatGPT and a panel of experts in the theoretical clinical decision-making process within the salivary gland clinic [20]. Additionally, Hoch et al. assessed ChatGPT’s accuracy across 2,576 theoretical questions covering 15 subspecialties of otolaryngology [21]. ChatGPT accurately resolved 57% of the questions, particularly single-choice questions [21], which are generally considered easier than multiple-choice questions where distracting information can increase the level of difficulty [22]. In fact, considering certain medical history information as important or not is a key human skill for determining the most plausible primary diagnosis [22].

Generally, there are two main sources of error: insufficient knowledge and inadequate information processing [22]. Specifically, the degree of complexity leads to errors due to incorrect information processing. Indeed, in our study, one of the complexities was identifying when a gastrostomy was necessary. We deliberately chose to exclude “gastrostomy” despite “chemotherapy” having a lower Kappa score (K = 0.28) because the indications for chemotherapy are well-established in the literature. We found that excluding gastrostomy indications, the agreement between the responses from ChatGPT-4 and those from the MOM increased from 0.35 to 0.48 for first-line treatment and from 0.56 to 0.78 for second-line treatment. This change can be attributed to the lack of clear consensus in the literature on prophylactic gastrostomy for patients with HNSCC [23-25]. The decision to implement a gastrostomy depends in many cases on weekly nutritional monitoring organized by dietitians. The threshold for placing a tube should be lower in patients who are more likely to need a PEG during their treatment, especially those with advanced disease of the oral cavity, oropharynx, and hypopharynx [1]. Notably, there is no consensus on the use of gastrostomy versus nasogastric tube feeding according to the European Society for Clinical Nutrition and Metabolism [1]. PEG, compared to nasogastric tubes, shows that body weight can be maintained similarly, the risk of tube dislodgement is lower, and the quality of life is probably better, but consideration must be given to the risks associated with the procedure of gastrostomy placement [26] and the increasing concern that gastrostomy placement leads to prolonged tube dependency and long-term dysphagia [27-29], while nasogastric tubes are associated with less dysphagia and faster weaning.

Furthermore, our study identified 52 patients for whom ChatGPT-4’s management recommendations differed from those of the MOM as well as the NCCN guidelines for the relevant year. Among these patients, seven cases were noted where ChatGPT-4 recommended radio-chemotherapy despite clear medical contraindications such as renal or cardiac failure. This highlights ChatGPT-4’s limitation in incorporating comorbidities and specific contraindications into its treatment advice. For another seven patients, ChatGPT-4 demonstrated limitations in managing geriatric oncology patients by proposing radio-chemotherapy treatment. However, the updated 2021 MACH-NC meta-analysis [30] suggests that there is no long-term benefit from chemotherapy for patients over 70 years old with advanced, non-metastatic head and neck cancer. Therefore, for these patients, the MOM recommended exclusive radiotherapy. This proves that ChatGPT-4 does not consider age-specific recommendations and long-term benefits.

Moreover, we observed that ChatGPT-4 is unable to estimate whether an SS is feasible. For instance, consider a patient with a p16-negative cT3N2cM0 SCC of the tonsil who initially received radio-chemotherapy, a treatment approach endorsed by both the MOM and ChatGPT-4. Post-treatment evaluations revealed that the cancer had not been fully eradicated, showing multifocal masses in the oropharynx. While these masses were deemed inoperable by the MOM’s surgeons, ChatGPT-4 suggested surgery as a viable treatment option. This situation prompts the question: Could we train AI systems, similar to convolutional neural networks, which have been trained on over 129,450 images of skin lesions to classify skin cancers with proficiency comparable to dermatologists, to assess the operability of lesions based on imaging data? [31,32].

Additionally, we found that by incorporating the NCCN guidelines, which in some cases offer multiple therapeutic options for the same condition, the agreement of responses improved from moderate agreement (Kappa score of 0.48) to substantial agreement (Kappa score of 0.72) for first-line treatments (while excluding gastrostomy indications). This suggests that with the broadening array of treatment choices, the complexity of decision-making for cancer patients has increased [2]. In this context, multidisciplinary oncological meetings play a crucial role in enhancing patient management by combining various expert opinions to devise a treatment strategy that is not only effective in treating cancer but also, in cases of incurable diseases, focused on significantly improving quality of life [2,33]. These variations in recommendations and practices can contribute to the discrepancies observed in our study between the decisions made by the MOM and those suggested by ChatGPT-4. As an AI tool based on published data and research, it may reflect a diversity of opinions that do not necessarily align with the specific practices prevailing in a particular hospital setting.

## Conclusions

This study highlights the challenges associated with integrating AI into oncological care. Although ChatGPT-4 offers considerable potential to improve diagnostic accuracy and optimize treatment plans in theory, its application in real clinical practice requires a deep understanding of its current limitations, particularly its ability to integrate complex clinical factors and adapt to updates in medical guidelines. To make progress, it is essential to continue evaluating and refining AI models so that they can effectively support healthcare professionals in making realistic and nuanced clinical decisions.

## References

[1] Arends J, Bachmann P, Baracos V (2017). ESPEN guidelines on nutrition in cancer patients. Clin Nutr.

[2] Lamb B, Green JS, Vincent C, Sevdalis N (2011). Decision making in surgical oncology. Surg Oncol.

[3] Arboleda LP, Neves AB, Kohler HF (2023). Overview of glottic laryngeal cancer treatment recommendation changes in the NCCN guidelines from 2011 to 2022. Cancer Rep (Hoboken).

[4] Wierzbicka M, Napierała J (2017). Updated National Comprehensive Cancer Network guidelines for treatment of head and neck cancers 2010-2017. Otolaryngol Pol.

[5] Jie Z, Zhiying Z, Li L (2021). A meta-analysis of Watson for Oncology in clinical application. Sci Rep.

[6] Temsah MH, Jamal A, Aljamaan F, Al-Tawfiq JA, Al-Eyadhy A (2023). ChatGPT-4 and the Global Burden of Disease Study: advancing personalized healthcare through artificial intelligence in clinical and translational medicine. Cureus.

[7] Lechien JR, Georgescu BM, Hans S, Chiesa-Estomba CM (2024). ChatGPT performance in laryngology and head and neck surgery: a clinical case-series. Eur Arch Otorhinolaryngol.

[8] Salvagno M, Taccone FS, Gerli AG (2023). Can artificial intelligence help for scientific writing?. Crit Care.

[9] Santer M, Kloppenburg M, Gottfried TM (2022). Current applications of artificial intelligence to classify cervical lymph nodes in patients with head and neck squamous cell carcinoma-a systematic review. Cancers (Basel).

[10] Hirotaka T, Shannon LW, Hiroyuki T (2024). Diagnostic performance comparison between generative AI and physicians: a systematic review and meta-analysis [PREPRINT]. medRxiv.

[11] Tessler I, Wolfovitz A, Alon EE, Gecel NA, Livneh N, Zimlichman E, Klang E (2024). ChatGPT's adherence to otolaryngology clinical practice guidelines. Eur Arch Otorhinolaryngol.

[12] NCCN Clinical Practice Guidelines in Oncology (NCCN Guidelines®) - Head and Neck Cancers Version 1.2016. https://oralcancerfoundation.org/wp-content/uploads/2016/09/head-and-neck.pdf.

[13] Adelstein D, Gillison ML, Pfister DG (2017). NCCN Guidelines Insights: Head and Neck Cancers, Version 2.2017. J Natl Compr Canc Netw.

[14] Colevas AD, Yom SS, Pfister DG (2018). NCCN Guidelines Insights: Head and Neck Cancers, Version 1.2018. J Natl Compr Canc Netw.

[15] Pfister DG, Spencer S, Adelstein D (2020). Head and Neck Cancers, Version 2.2020, NCCN Clinical Practice Guidelines in Oncology. J Natl Compr Canc Netw.

[16] NCCN Clinical Practice Guidelines in Oncology (NCCN Guidelines ®) - Head and Neck Cancers Version 3.2021. https://www.poijaya.org/wp-content/uploads/2021/08/head-and-neck.pdf.

[17] McHugh ML (2012). Interrater reliability: the kappa statistic. Biochem Med (Zagreb).

[18] Kung TH, Cheatham M, Medenilla A (2023). Performance of ChatGPT on USMLE: potential for AI-assisted medical education using large language models. PLOS Digit Health.

[19] Kuşcu O, Pamuk AE, Sütay Süslü N, Hosal S (2023). Is ChatGPT accurate and reliable in answering questions regarding head and neck cancer?. Front Oncol.

[20] Chiesa-Estomba CM, Lechien JR, Vaira LA (2024). Exploring the potential of Chat-GPT as a supportive tool for sialendoscopy clinical decision making and patient information support. Eur Arch Otorhinolaryngol.

[21] Hoch CC, Wollenberg B, Lüers JC (2023). ChatGPT's quiz skills in different otolaryngology subspecialties: an analysis of 2576 single-choice and multiple-choice board certification preparation questions. Eur Arch Otorhinolaryngol.

[22] Braun LT, Lenzer B, Fischer MR, Schmidmaier R (2019). Complexity of clinical cases in simulated learning environments: proposal for a scoring system. GMS J Med Educ.

[23] Ahmed KA, Samant S, Vieira F (2005). Gastrostomy tubes in patients with advanced head and neck cancer. Laryngoscope.

[24] McClelland S 3rd, Andrews JZ, Chaudhry H, Teckie S, Goenka A (2018). Prophylactic versus reactive gastrostomy tube placement in advanced head and neck cancer treated with definitive chemoradiotherapy: a systematic review. Oral Oncol.

[25] Nugent B, Lewis S, O'Sullivan JM (2013). Enteral feeding methods for nutritional management in patients with head and neck cancers being treated with radiotherapy and/or chemotherapy. Cochrane Database Syst Rev.

[26] Grant DG, Bradley PT, Pothier DD, Bailey D, Caldera S, Baldwin DL, Birchall MA (2009). Complications following gastrostomy tube insertion in patients with head and neck cancer: a prospective multi-institution study, systematic review and meta-analysis. Clin Otolaryngol.

[27] Langmore S, Krisciunas GP, Miloro KV, Evans SR, Cheng DM (2012). Does PEG use cause dysphagia in head and neck cancer patients?. Dysphagia.

[28] Corry J, Poon W, McPhee N (2008). Randomized study of percutaneous endoscopic gastrostomy versus nasogastric tubes for enteral feeding in head and neck cancer patients treated with (chemo)radiation. J Med Imaging Radiat Oncol.

[29] Mekhail TM, Adelstein DJ, Rybicki LA (20011). Enteral nutrition during the treatment of head and neck carcinoma: is a percutaneous endoscopic gastrostomy tube preferable to a nasogastric tube?. Cancer.

[30] Lacas B, Carmel A, Landais C (2021). Meta-analysis of chemotherapy in head and neck cancer (MACH-NC): an update on 107 randomized trials and 19,805 patients, on behalf of MACH-NC Group. Radiother Oncol.

[31] Esteva A, Kuprel B, Novoa RA, Ko J, Swetter SM, Blau HM, Thrun S (2017). Dermatologist-level classification of skin cancer with deep neural networks. Nature.

[32] Haenssle HA, Fink C, Schneiderbauer R (2018). Man against machine: diagnostic performance of a deep learning convolutional neural network for dermoscopic melanoma recognition in comparison to 58 dermatologists. Ann Oncol.

[33] Jerusalem G, Coucke P (2011). Apport de la Consultation Oncologique Multidisciplinaire dans le choix des options thérapeutiques. Rev Med Liège.

